# Antibody effector function is regulated by a combination of adaptive and innate signals

**DOI:** 10.1186/1742-4690-9-S2-O37

**Published:** 2012-09-13

**Authors:** A Mahan, K Dionne, J Eusebio, G Alter

**Affiliations:** 1 Harvard University, Charlestown, MA, USA; 2 Ragon Institute, Charlestown, MA, USA

## Background

The Fc region of IgG contains a single N-glycosylation site, which is known to be important for effecting immune activation through interaction with Fc receptors and complement molecules. Changes to the structure of these sugars can thus be very influential in determining the specific effector function of individual antibodies. Whereas therapeutic antibodies can be produced with particular effector functions in vitro, little is known regarding how glycosylation is regulated in primary B cells.

## Methods

Purified B cells were stimulated with a variety of synthetic TLR ligands alone or in combination with soluble CD40L, anti-IgG, anti-IgM, or supernatants derived from stimulated antigen presenting cells. After 16 hours, quantitative RT-PCR was used to determine expression of genes known to be specifically involved in IgG N-glycan synthesis.

## Results

We found that the expression of glycosylation genes is significantly impacted both by specific TLR stimulation alone and in combination with adaptive signals received either through the B cell receptor or CD40. Specifically, virus-derived stimuli that activate TLR 7, 8 or 9 can significantly decrease the expression of galactose adding enzymes, whereas TLR 9 stimulation in combination with CD40 stimulation decreases the expression of both sialic acid– or GlcNac-adding enzymes. These changes in expression result in the production of antibodies with Fc glycan structures with increased NK cell– and monocyte-recruiting capabilities.

## Conclusion

Overall, these results are the first to show that the production of antibodies with specific effector functions can be regulated by external stimuli, including both innate and adaptive immune signals, suggesting that antibodies with specific, strong effector functions can be induced in vivo following vaccination.

